# Perceived environmental support for values education and mental health among Chinese aviation university students: the mediating role of psychological resilience and the moderating role of flight training duration

**DOI:** 10.3389/fpsyg.2026.1910119

**Published:** 2026-08-05

**Authors:** Yingnan Guo, Zhi Guo, Zongdi Yin

**Affiliations:** 1 School of Marxism, Civil Aviation University of China, Tianjin, China; 2 Tianjin Jinhang Technical Institute of Physics, Tianjin, China

**Keywords:** perceived environmental support, aviation university students, mental health, psychological resilience, values education, flight training duration

## Abstract

**Objective:**

This study aimed to explore the relationship between perceived environmental support for value education (PESVE) and mental health among civil aviation flight university students, and to examine the mediating role of psychological resilience and the moderating effect of flight training duration.

**Methods:**

A cross-sectional survey was conducted among 1,126 flight technology students from the Civil Aviation University of China using the stratified cluster sampling method. Participants completed the Self-developed Scale of Value Education Integration (SVEI), the Chinese version of the Depression Anxiety Stress Scale (DASS-21), and the Connor-Davidson Resilience Scale (CD-RISC). Structural equation modeling (SEM) was used to test the mediating effect of psychological resilience, and a multi-group analysis examined the moderating role of flight training duration.

**Results:**

The mean scores were as follows: PESVE (52.80 ± 10.65), resilience (73.46 ± 14.32), and DASS-21 total (37.50 ± 22.86). PESVE was positively correlated with resilience (r = 0.46, *p* < 0.01) and negatively correlated with DASS-21 total (r = −0.41, *p* < 0.01). Resilience was negatively correlated with DASS-21 total (r = −0.52, *p* < 0.01). SEM showed a good fit (χ^2^/df = 2.63, CFI = 0.95, RMSEA = 0.055). The indirect effect of resilience was −0.24 (95% CI: −0.31 to −0.18), accounting for 42.1% of the total effect. The multi-group analysis revealed that the path coefficient from PESVE to resilience was significantly higher for students with >100 flight hours (*β* = 0.58) than for those with <50 flight hours (β = 0.35).

**Conclusion:**

Perceived environmental support for value education is associated with improved mental health among flight university students, partially through the enhancement of psychological resilience, and this association becomes stronger with increased professional training. A dual-core (value-psychology) collaborative mechanism in curriculum, activities, and management is recommended for holistic student development.

## 1 Introduction

The mental health of university students is a global concern, particularly for those in high-stress professional training programs such as civil aviation pilots. Flight students face unique stressors—including academic pressure, flight training demands, and future occupational responsibilities—that can compromise their psychological well-being and ultimately affect public flight safety [Li and Xu, 2025; International Air Transport Association (IATA), 2022]. In China, the Civil Aviation Administration has explicitly emphasized integrating value education (often referred to as “ideological and political education”) into mental health promotion for flight students, stating that “cultivating psychological resilience and optimistic qualities” is essential for professional development (Civil Aviation Administration of China, 2025). However, research on how value education relates to mental health and the mechanisms underlying this relationship remains limited.

Conceptualization of perceived environmental support for value education:

The central construct of this study—perceived environmental support for value education (PESVE)—refers to students’ subjective perception of the extent to which the educational environment, including formal curricula, extracurricular activities, and institutional management services, provides consistent and meaningful value education content. This construct is grounded in Bronfenbrenner’s ecological systems theory (Bronfenbrenner, 1979), which posits that individuals’ development is shaped by their perceptions of and interactions with multiple layers of their environment—the microsystem (immediate settings such as classrooms and training environments), mesosystem (interactions among microsystems), exosystem (indirect environmental influences), and macrosystem (cultural and societal values). In the context of flight training, PESVE captures students’ perceived exposure to value-related content across three key environmental layers: (a) curriculum integration—value content embedded in formal coursework, (b) activity integration—values transmitted through practical training and extracurricular experiences, and (c) management-service integration—values reflected in institutional policies and daily support services. This tripartite framework draws upon the theoretical work of Shen (2016), whose theory on the effectiveness of ideological and political education emphasizes that the impact of value education depends on the “alignment” between educational content and student perception across multiple delivery channels. It is also influenced by Yu (2020), whose theory of mental health education highlights the integration of value guidance with psychological support as a “values-psychology” dual-core collaborative mechanism.

Previous studies have shown that supportive educational environments are associated with enhanced psychological resilience—the ability to recover from adversity—which in turn relates to reduced symptoms of depression, anxiety, and stress. It should be noted that Connor and Davidson (2003) and Yu and Zhang (2007) provided the psychometric foundation for the CD-RISC resilience measure rather than directly examining educational environments; their studies establish the methodological basis for assessing resilience in the present study. Resilience has been identified as a key factor linking environmental support and mental health outcomes in general student populations.

For instance, Ye et al. (2018) found that social support moderated the mediating effect of resilience between perceived stress and depression. Similarly, Hao and Lei (2007) demonstrated that resilience acts as a buffer against academic pressure and daily life stressors. Nevertheless, few studies have examined this mediation pathway among flight students, who are characterized by rigorous training and high occupational demands.

Moreover, the potential moderating role of flight training duration (i.e., cumulative flight hours) in the relationship between value education and resilience has not been investigated. According to embodied cognition theory (Ye, 2010), practical experience may strengthen the internalization of values, suggesting that the association between value education and resilience may become stronger as students gain more flight training experience. Recent international research has increasingly emphasized the role of learning and training in supporting emotional resilience within high-demand professional contexts (Sedlačík et al., 2026), the pathways through which autonomous supportive teaching environments foster academic resilience (Qian and Masroor, 2026), and the chain mediating effects of self-efficacy and psychological resilience between physical activity and academic stress (Leng et al., 2025). These studies collectively underscore the importance of examining both individual psychological resources and environmental supports in understanding student mental health.

**Figure 1:**
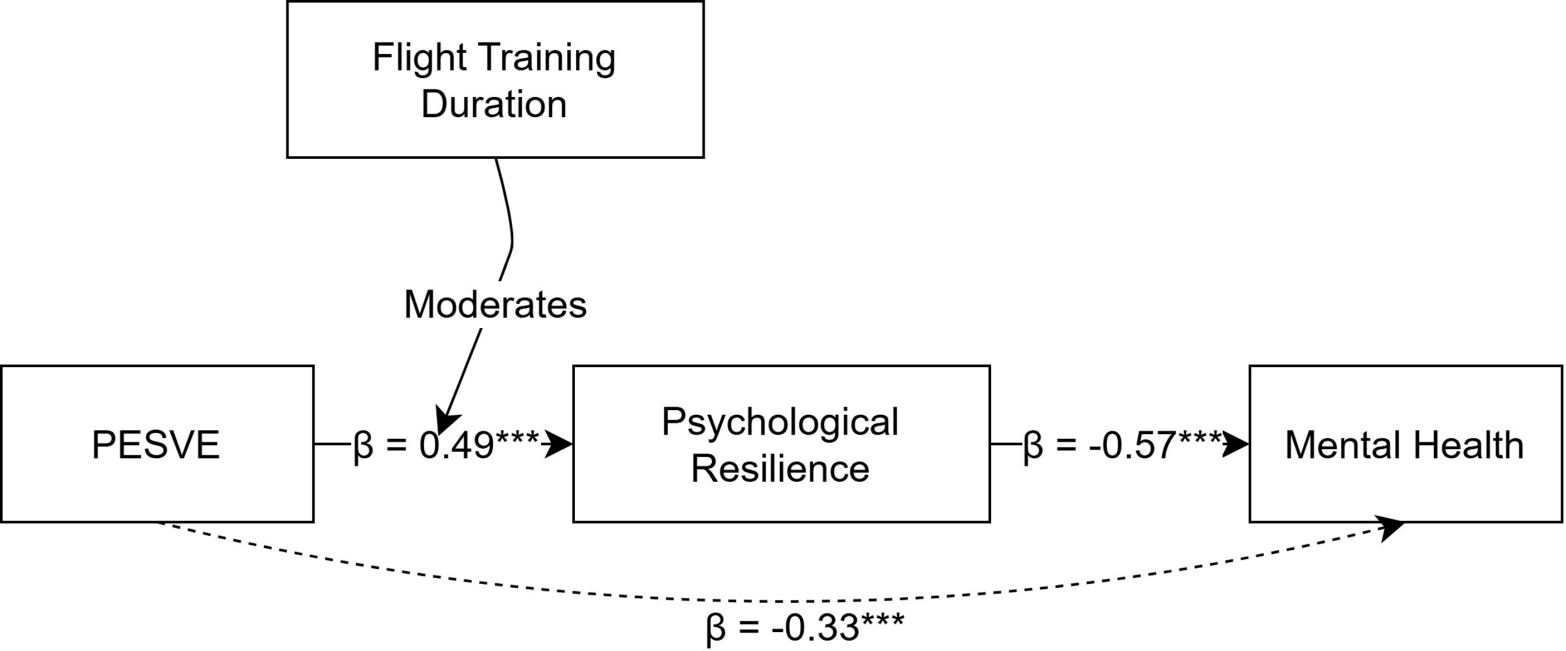
Conceptual model with mediation and moderation.

Therefore, the present study aims to (1) test the mediating role of psychological resilience in the relationship between perceived environmental support for value education (PESVE) and mental health (depression, anxiety, and stress) among Chinese flight university students; and (2) examine whether flight training duration moderates the PESVE–resilience pathway. We propose a moderated mediation model (Figure 1) and hypothesize that:

*H1:* PESVE is positively associated with psychological resilience.

*H2:* Psychological resilience is negatively associated with mental health problems.

*H3:* Resilience mediates the relationship between PESVE and mental health.

*H4:* Flight training duration moderates the PESVE–resilience link, such that the effect is stronger for students with longer training hours.

## 2 Materials and methods

### 2.1 Participants and procedure

A cross-sectional survey was conducted between March and May 2025 at the Civil Aviation University of China (CAUC). Using stratified cluster sampling by grade and class, we recruited 1,200 flight technology students. Inclusion criteria included: (1) currently enrolled in the flight technology program; (2) provided informed consent. Exclusion criteria included: (1) diagnosed with severe mental disorders and (2) experienced major negative life events in the past 6 months. After excluding invalid responses (*n* = 54; 4.5%—based on completion time <300 s or >1800 s, patterned responses, or failed attention check), 1,126 valid questionnaires remained (effective rate 93.8%). Sample characteristics: freshman 25.6%, sophomore 26.2%, junior 24.9%, and senior 23.3%; urban origin 57.0%; only child 52.1%; flight training hours: <50 h (32.4%), 50–100 h (35.3%), and >100 h (32.3%). The study was approved by the Institutional Review Board of CAUC (approval no. CAUC-IRB-2025-038). All participants signed informed consent.

Table 1 presents the demographic characteristics of the sample.

**Table 1:** Demographic characteristics of participants (*N* = 1,126).

Characteristic	Category	*n*	%
Grade	Freshman	288	25.6
	Sophomore	295	26.2
	Junior	280	24.9
	Senior	263	23.3
Hometown	Urban	642	57.0
	Rural	484	43.0
Only child	Yes	587	52.1
	No	539	47.9
Flight hours	<50 h	365	32.4
	50–100 h	398	35.3
	>100 h	363	32.3

### 2.2 Measures

#### 2.2.1 Perceived environmental support for value education (PESVE)

We have developed the Scale of Value Education Integration (SVEI) based on the theoretical frameworks of Shen (2016) and Yu (2020), as well as the specific context of civil aviation training. The scale development follows standardized procedures:

1. Item generation and content validity. Based on the theoretical frameworks—Shen's (2016) theory of ideological and political education effectiveness and Yu's (2020) mental health education theory—an initial pool of 20 items was generated across three dimensions: curriculum integration (value content embedded in coursework), activity integration (values transmitted through practical training and extracurricular activities), and management-service integration (values reflected in institutional policies and support services). Expert consultation was conducted with 10 experts (5 in values education, 3 in mental health, and 2 flight instructors) using a two-round Delphi to evaluate item relevance and clarity. Content validity indices (I-CVI) ranged from 0.80 to 1.00, and the scale-level content validity index (S-CVI/Ave) was 0.92, indicating excellent content validity.
2. Pilot testing and item analysis. Pilot testing *N* = 180 was conducted with item analysis (critical ratio, item-total correlation) and exploratory factor analysis (principal component, Promax rotation). Items with factor loading <0.5 or cross-loading were deleted (3 items removed).
3. Final scale. The final 15-item scale (5 per dimension) uses a 5-point Likert scale (1 = completely disagree, 5 = completely agree), with a total score range of 15–75. Representative items include: Curriculum integration—"My coursework regularly incorporates discussions of professional ethics and values”; Activity integration—"Flight training activities emphasize the importance of responsibility and teamwork”; Management-service integration—"The university’s student management policies reflect core values such as integrity and dedication.” In the current sample, Cronbach’s *α* for the total scale was 0.91; subscale αs ranged from 0.83 to 0.87.
4. Construct validity. Confirmatory factor analysis (CFA) tested a second-order factor model in which the three first-order factors (curriculum, activity, management-service integration) loaded onto a second-order PESVE factor. The model showed good fit: χ^2^/df = 2.89, CFI = 0.94, TLI = 0.92, RMSEA = 0.065. Second-order factor loadings were 0.85, 0.92, and 0.88 (all *p* < 0.001), supporting the use of the total score as an indicator of PESVE. Average variance extracted (AVE) ranged from 0.51 to 0.58, and composite reliability (CR) ranged from 0.84 to 0.88, indicating adequate convergent and discriminant validity.

#### 2.2.2 Mental health

The Chinese version of the Depression Anxiety Stress Scales-21 (DASS-21) (Gong et al., 2010) was used. It has three subscales (depression, anxiety, stress), each with seven items, rated on a 4-point scale (0 = never, 3 = always). Subscale scores are multiplied by 2, yielding ranges from 0 to 42. Higher scores indicate more severe symptoms. In this study, Cronbach’s *α* was 0.92 for the total scale and 0.88, 0.84, and 0.86 for the depression, anxiety, and stress subscales, respectively.

#### 2.2.3 Psychological resilience

The Connor-Davidson Resilience Scale (CD-RISC) Chinese version (Yu and Zhang, 2007) was used. It contains 25 items rated on a 5-point scale (0 = never, 4 = almost always), with a total score range of 0–100. Higher scores indicate greater resilience. Cronbach’s α was 0.93.

#### 2.2.4 Flight training duration

Self-reported cumulative flight hours, categorized as <50 h, 50–100 h, and >100 h. These cutoffs correspond to key milestones in Chinese civil aviation flight training—50 h typically marks solo flight completion, and 100 h marks Private Pilot License (PPL) acquisition—as specified in the CAAC flight training syllabus (MD-141-FS-101).

### 2.3 Statistical analysis

Descriptive statistics, reliability, and correlations were computed using SPSS 26.0. Harman’s single-factor test and the unmeasured latent method factor (ULMC) approach were used to assess common method bias.

Prior to structural equation modeling, key analytical assumptions were examined: normality (skewness <|2|, kurtosis <|7| for all variables), outliers (no cases with standardized Z > 3.29), linearity (scatterplot matrices indicate linear relationships), multicollinearity (VIF < 2.5), and missing data (MCAR test was non-significant; complete-case analysis was used).

Structural equation modeling (SEM) was performed in Mplus 8.7 to test the mediation model. Prior to testing the structural model, the measurement model, including all latent variables, was evaluated and showed good fit: χ^2^/df = 2.71, CFI = 0.96, TLI = 0.95, RMSEA = 0.052, SRMR = 0.044, with all factor loadings significant (*p* < 0.001) and standardized loadings ranging from 0.52 to 0.84. Model fit was evaluated using χ^2^/df (<3 ideal, <5 acceptable), CFI/TLI (>0.95 ideal, >0.90 acceptable), RMSEA, and SRMR (<0.05 ideal, <0.08 acceptable). The indirect effect was tested using bias-corrected bootstrapping with 5,000 resamples; 95% confidence intervals (CIs) that did not contain zero were considered significant. Explained variance (R^2^) for endogenous variables and effect sizes (Cohen’s f^2^) were also reported.

Multi-group SEM was used to examine moderation by flight training duration. Measurement invariance (configural, metric, scalar) was tested using ΔCFI < −0.01 and ΔRMSEA <0.015 as criteria (Cheung and Rensvold, 2002). Then the structural path “PESVE → resilience” was constrained to be equal across groups; a significant Δχ^2^ test indicated moderation.

All tests were two-tailed with *α* = 0.05. In initial analyses, we controlled for demographic variables (gender, grade, urban/rural origin, and only-child status) and found that these did not significantly affect the main path coefficients (changes <0.03) or model fit (ΔCFI <0.005). Thus, the final model is reported without these controls for parsimony.

## 3 Results

### 3.1 Common method bias and descriptive statistics

Harman’s single-factor test extracted 10 factors with eigenvalues >1; the first factor explained 28.7% of variance (<40% cutoff), indicating no severe common method bias. The ULMC approach further supported this conclusion: adding a common method factor to the CFA model resulted in limited improvement (ΔCFI = 0.008, ΔRMSEA = 0.004), and all substantive factor loadings remained significant.

Table 2 presents descriptive statistics. The mean PESVE score was 52.80 ± 10.65; the mean resilience was 73.46 ± 14.32; the mean DASS-21 total was 37.50 ± 22.86. Prevalence rates of mild or above depression, anxiety, and stress symptoms were 15.2, 18.7, and 24.5%, respectively, indicating notable stress problems.

**Table 2:** Descriptive statistics of the main study variables (*N* = 1,126).

Variable	Dimension/total	Items	Theoretical range	M	SD	Cronbach’s α
PESVE	Curriculum integration	5	5–25	16.65	3.89	0.85
	Activity integration	5	5–25	18.40	3.41	0.87
	Management-service integration	5	5–25	17.75	3.56	0.83
	Total	15	15–75	52.80	10.65	0.91
Resilience (CD-RISC)	Total	25	0–100	73.46	14.32	0.93
DASS-21	Depression (×2)	7	0–42	10.24	11.34	0.88
	Anxiety (×2)	7	0–42	11.96	11.78	0.84
	Stress (×2)	7	0–42	15.30	12.02	0.86
	**Total (sum of raw scores)**	**21**	**0–126**	**37.50**	**22.86**	**0.92**

DASS-21 subscale scores are multiplied by 2 as per standard scoring. The total score shown is the sum of raw scores before multiplication. Bolded values represent the total scores for each respective scale (PESVE, Resilience, and DASS-21). These totals reflect the overall level of the construct and are used in the primary analyses. For DASS-21, the total score is the sum of raw item scores before multiplication by 2; the subscale scores (Depression, Anxiety, Stress) are multiplied by 2 as per standard scoring guidelines.

### 3.2 Correlations

Pearson’s correlations (Table 3) showed that PESVE total and its subscales were positively correlated with resilience (r = 0.31–0.46, *p* < 0.01) and negatively correlated with DASS-21 total r = −0.28 to −0.41, *p* <0.01. Resilience was negatively correlated with DASS-21 total r = −0.52, *p* < 0.01. These results supported further mediation analysis.

**Table 3:** Pearson’s correlation matrix (*N* = 1,126).

Variable	1	2	3	4	5	6	7	8
1. PESVE – Curriculum	1							
2. PESVE – Activity	0.68**	1						
3. PESVE – Management	0.62**	0.71**	1					
4. PESVE – Total	0.88**	0.91**	0.89**	1				
5. Resilience – Total	0.31**	0.42**	0.40**	0.46**	1			
6. DASS – Depression	−0.28**	−0.35**	−0.33**	−0.38**	−0.48**	1		
7. DASS – Anxiety	−0.30**	−0.36**	−0.34**	−0.39**	−0.44**	0.72**	1	
8. DASS – Stress	−0.33**	−0.39**	−0.37**	−0.41**	−0.52**	0.65**	0.69**	1
9. DASS – Total	−0.33**	−0.39**	−0.37**	−0.41**	−0.52**	0.89**	0.90**	0.88**

**p < 0.01.

### 3.3 Mediating role of psychological resilience

The SEM model demonstrated good fit: χ^2^/df = 2.63, CFI = 0.95, TLI = 0.94, RMSEA = 0.055 (90% CI: 0.050–0.060), SRMR = 0.048. The model explained 24.0% of the variance in resilience (R^2^ = 0.240) and 42.3% of the variance in mental health problems (R^2^ = 0.423).

**Figure 2:**
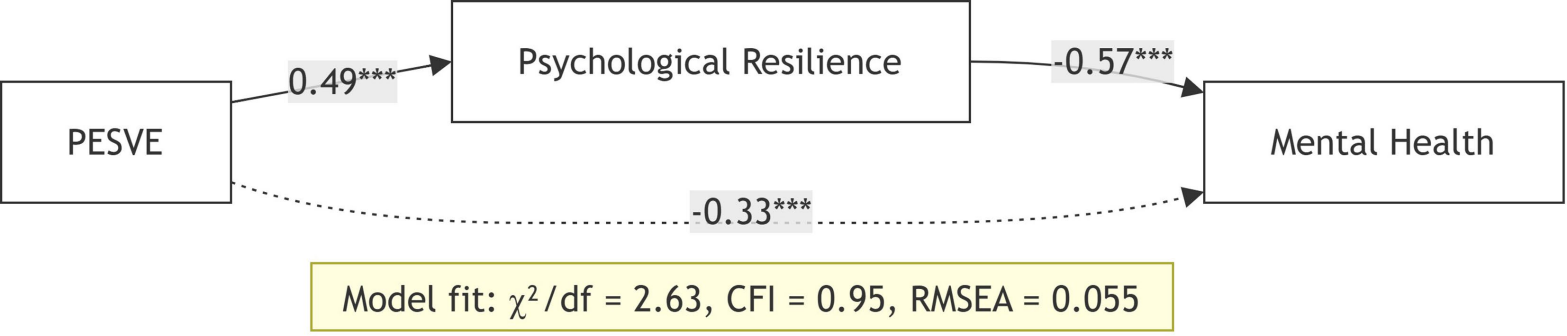
Mediation model of perceived environmental support for values education (PESVE) on mental health through psychological resilience. Standardized path coefficients are shown. ***p < 0.001. Model fit indices: χ^2^/df = 2.63, CFI = 0.95, RMSEA = 0.055.

Path coefficients (Figure 2) showed that PESVE positively predicted resilience (*β* = 0.49, *p* < 0.001; Cohen’s f^2^ = 0.32); resilience negatively predicted mental health problems (β = −0.57, *p* < 0.001; Cohen’s f^2^ = 0.48); PESVE also had a direct negative association with mental health problems (β = −0.33, *p* < 0.001).

Bootstrap analysis (Table 4) indicated that the indirect effect via resilience was −0.24 (95% CI: −0.31 to −0.18), accounting for 42.1% of the total effect (−0.57). Thus, resilience partially mediated the PESVE–mental health relationship.

**Table 4:** Bootstrap analysis of the mediating effect of resilience.

Effect type	Effect (β)	Boot SE	95% CI	Proportion (%)
Direct effect (PESVE → mental health)	−0.33	0.04	[−0.41, −0.25]	57.9
Indirect effect (via resilience)	−0.24	0.03	[−0.31, −0.18]	42.1
Total effect	−0.57	0.05	[−0.67, −0.47]	100.0

Bias-corrected bootstrapping with 5,000 resamples. CI = confidence interval.

### 3.4 Moderating effect of flight training duration

Measurement of invariance across the three flight duration groups was supported. Table 5 presents the invariance testing results.

**Table 5:** Measurement invariance testing across flight training duration groups.

Model	χ^2^	df	CFI	RMSEA	SRMR	ΔCFI	ΔRMSEA
Configural invariance	486.32	213	0.951	0.058	0.046	—	—
Metric invariance	501.87	229	0.948	0.057	0.049	−0.003	−0.001
Scalar invariance	522.15	245	0.944	0.058	0.051	−0.004	+0.001

ΔCFI and ΔRMSEA values are within acceptable ranges (ΔCFI > − 0.01, ΔRMSEA <0.015), supporting measurement invariance across groups.

**Figure 3:**
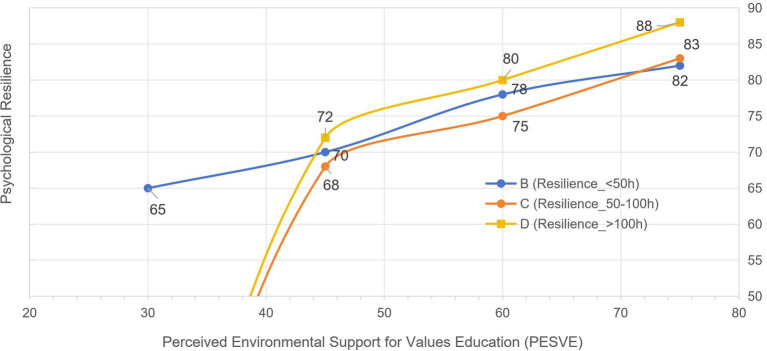
Moderating effect of flight training duration on the relationship between perceived environmental support for values education (PESVE) and psychological resilience. Lines represent groups based on training hours: <50 h (blue), 50–100 h (orange), and >100 h (yellow).

The unconstrained model (M0) showed a good fit. Constraining the PESVE→resilience path to be equal across groups (M1) resulted in a significant Δχ^2^(2) = 8.76, *p* = 0.012, ΔCFI = -0.012, indicating that the path coefficient differed significantly by training duration. Specifically, Figure 3 for low-duration group (<50 h), β = 0.35 (*p* < 0.001); medium-duration (50–100 h), β = 0.47 (*p* < 0.001); high-duration (>100 h), β = 0.58 (*p* < 0.001). Thus, the association between perceived value of education support and resilience strengthened as flight training experience increased. A supplementary analysis treating flight hours as a continuous variable using latent moderated structural equation modeling (LMS) yielded a consistent interaction effect (β = 0.18, *p* < 0.01), confirming that the categorical analysis did not distort the true moderating relationship.

## 4 Discussion

This study examined the relationship between perceived environmental support for value education (PESVE) and mental health among Chinese flight university students, focusing on the mediating role of psychological resilience and the moderating role of flight training duration. Our findings support all four hypotheses and provide both theoretical and practical contributions.

First, consistent with H1 and H2, PESVE was positively associated with resilience, and resilience was negatively associated with mental health problems. These results align with previous research on general student populations (e.g., Ye et al., 2018; Hao and Lei, 2007) and extend them to the specific context of flight students. Value education—conveying ideals, professional ethics, and collectivism—may provide students with a meaningful framework that helps transcend personal difficulties, thereby enhancing self-efficacy and an optimistic explanatory style (Seligman, 2011). Recent international research further supports this interpretation: Putri (2024) found that social support is a critical resource for educational resilience among students facing academic challenges, while Qian and Masroor (2026) demonstrated that autonomous supportive teaching environments foster academic resilience by enhancing student engagement and self-determination.

Second, resilience partially mediated the PESVE–mental health link (H3), with an indirect effect accounting for 42.1% of the total effect. This suggests that value education not only has a direct negative association with negative emotions but also operates through strengthening psychological resilience—a more sustainable mechanism for long-term mental health promotion. The finding underscores the importance of embedding resilience-building components within value education curricula. Leng et al. (2025) similarly demonstrated that psychological resilience serves as a chain mediator between physical activity and academic stress among Chinese adolescents, highlighting the generalizable role of resilience in educational settings.

Third, flight training duration moderated the PESVE→resilience pathway (H4). The association between perceived value education support and resilience was stronger among students with more flight hours (>100 h) than among those with fewer hours (<50 h). This is consistent with embodied cognition theory (Ye, 2010): as students engage in authentic flight operations, emergency handling, and teamwork, abstract values (e.g., “respect for life,” “responsibility”) become concretized through personal experience. The internalization of values is thereby deepened, translating external norms into stable psychological traits (Gu, 2017). This finding highlights the importance of integrating value education with professional practice: the synergy between cognitive learning and experiential learning amplifies the benefits. Sedlačík et al. (2026) found analogous patterns in security forces training, where learning and practical training synergistically supported emotional resilience.

### 4.1 Practical implications

Based on these findings, we propose a “dual-core (value-psychology), three-dimensional (curriculum, activity, management)” collaborative mechanism for holistic student development. Specific strategies include:

#### 4.1.1 Curriculum dimension

Developing stress management modules that integrate aviation professional ethics, embedding value discussions into flight theory courses, and creating case-based learning materials that link professional values to mental health outcomes.

#### 4.1.2 Activity dimension

Designing team-based psychological training using crew resource management (CRM) principles, organizing value-oriented extracurricular activities (e.g., volunteer service, ethics forums), and incorporating resilience-training components into flight simulation exercises.

#### 4.1.3 Management dimension

Establishing joint assessment systems that monitor both ideological and mental health status, training flight instructors to recognize and respond to students’ psychological needs, and creating institutional policies that explicitly link value education with mental health support services.

Institutions training high-stress professionals (e.g., pilots, military personnel, seafarers) should embed value education within authentic practice contexts to maximize its protective associations with mental health.

### 4.2 Limitations and future directions

Several limitations should be acknowledged.

First, the cross-sectional design precludes causal inferences. Reverse causation is equally plausible—students with better mental health or higher resilience may perceive their educational environment more positively. Future longitudinal or intervention studies are needed to establish temporal precedence and causal direction.

Second, although the sample was drawn from the Civil Aviation University of China—the primary institution for civil aviation pilot training in China—and included students across all four grades with varying flight training hours, the single-site nature limits generalizability to other aviation training institutions in China or abroad, as well as to other high-stress professional training contexts (e.g., military academies, maritime institutes, and medical residency programs). Cultural differences in value education approaches—particularly between collectivist and individualist educational systems—may also affect its applicability. Cross-cultural replications are needed.

Third, all measures were self-reported, which may introduce social desirability bias. Although both Harman’s single-factor test and the ULMC approach suggested that common method bias was not a severe threat, the exclusive reliance on self-report remains a limitation. Future research could incorporate peer or instructor ratings, behavioral observations, or physiological indicators.

Fourth, the SVEI, while demonstrating sound psychometric properties in this sample, requires further validation in independent samples, including test–retest reliability assessment and criterion-related validity assessment using external criteria (e.g., instructor ratings of value internalization).

Fifth, although we tested and found no significant confounding effects of demographic variables (gender, grade, urban/rural origin, and only-child status) on the main pathways, other unmeasured variables—such as socioeconomic status, prior psychiatric history, family support, recent stressful life events, and physical health—were not controlled for. Future studies should incorporate these variables to further rule out potential confounding.

Future research could also explore the mechanism using qualitative methods (e.g., interviews with flight instructors) and examine whether similar moderated mediation effects exist in other high-pressure professions.

## 5 Conclusion

Perceived environmental support for value education is associated with better mental health among flight university students, in part by enhancing psychological resilience. The beneficial association strengthens as students accumulate flight training hours, underscoring the importance of integrating value education with authentic professional practice. A collaborative mechanism that combines value guidance and psychological empowerment across curriculum, activities, and management is recommended to foster holistic development in high-stress professional training programs.

## Data Availability

The raw data supporting the conclusions of this article will be made available by the authors, without undue reservation.
